# Acoustic percolation switches enable targeted drug delivery controlled by diagnostic ultrasound

**DOI:** 10.1073/pnas.2423078122

**Published:** 2025-05-14

**Authors:** Maria Paulene Abundo, Anna T. Tifrea, Marjorie T. Buss, Pierina Barturen-Larrea, Zhiyang Jin, Dina Malounda, Mikhail G. Shapiro

**Affiliations:** ^a^Division of Chemistry and Chemical Engineering, California Institute of Technology, Pasadena, CA 91125; ^b^Andrew and Peggy Cherng Department of Medical Engineering, California Institute of Technology, Pasadena, CA 91125; ^c^Howard Hughes Medical Institute, Pasadena, CA 91125

**Keywords:** diffusion, hydrogels, ultrasound, drug delivery, gas vesicles

## Abstract

Delivering biomedicines to specific sites of disease using remote-controlled devices is a long-standing vision in biomedical research. However, most existing externally triggered delivery systems are based on complex micromachines that are controlled with electromagnetic waves and require custom external instrumentation. Here, we present a drug delivery platform based on a simple protein-containing hydrogel that can be both imaged and triggered to release drugs at specific locations using widely available diagnostic ultrasound devices. This technology is based on the addition of air-filled protein nanostructures called gas vesicles (GVs) to hydrogel delivery vehicles. While intact, GVs sterically block the release of drug payloads and allow the vehicle to be imaged with ultrasound. An increase in ultrasound pressure causes the collapse of GVs within the delivery vehicles at the desired anatomical location, instantly creating percolation channels in the hydrogel, massively increasing diffusivity, and leading to rapid drug release. Unlike previous ultrasound-actuated delivery approaches, both the imaging and release are performed using a simple diagnostic ultrasound probe ubiquitously available in clinical settings. We implement this concept by quantifying ultrasound-controlled drug diffusion and release in vitro and demonstrating image-guided protein delivery in vivo in the gastrointestinal (GI) tract following oral administration. We further validate this technology by using it to deliver anti-inflammatory antibodies to effectively treat a rat model of colitis. Targeted acoustic percolation switches (TAPS) open a conduit for local, image-guided drug delivery with a simple formulation and commonplace ultrasound equipment.

## Significance

The concept of remote-controlled devices that can enter the body and deliver biomedicines to specific sites of disease is a long-held vision in biomedical research. However, most existing devices for externally triggered delivery are based on complex micromachines that are controlled with electromagnetic waves and require custom external instrumentation. Here, we present a much simpler drug delivery platform comprising hydrogels that contain unique air-filled proteins allowing the gel to be both imaged and triggered to release drugs at specific locations using widely available ultrasound imaging devices. We validate this technology by delivering anti-inflammatory antibodies to effectively treat a rat model of colitis. Targeted acoustic percolation switches (TAPS) have the potential to bring image-guided drug delivery closer to everyday clinical practice.

The development of technologies for remote-controlled, targeted drug delivery represents a “holy grail” of biomedical research. When implemented successfully, these platforms allow therapeutics to reach specific anatomical locations in complex tissues such as the gastrointestinal (GI) tract, providing efficacy at the intended site of action while minimizing side effects. Ideally, remote-controlled drug delivery would be performed under image guidance, making it possible to monitor vehicle location, trigger release, and confirm payload delivery. However, executing these actions in vivo remains a challenge. Most existing approaches utilize complex micromachined devices triggered by electric (1) or magnetic fields (2, 3), which are challenging to focus in vivo, or by light (4, 5), which has limited tissue penetration (6). Moreover, these devices tend to be nondegradable (7), expensive to produce, difficult to miniaturize, and require dedicated external apparatus for monitoring and control (8, 9).

If instead it were possible to develop a drug delivery vehicle that could be tracked and triggered with diagnostic ultrasound, this would enable image-guided drug delivery using ubiquitous, low-cost instruments with deep penetration (>10 cm) and high resolution (<1 mm) (10). Moreover, if such vehicles could be produced via the simple mixing of biocompatible components and hydrogels that are commonly used in ingestible drug formulations (11, 12) such as chitosan (13, 14), alginate (15–17), acrylamide (18–20) polymers and their derivatives, this would accelerate their development for a broader range of clinical applications.

**Fig. 1.**
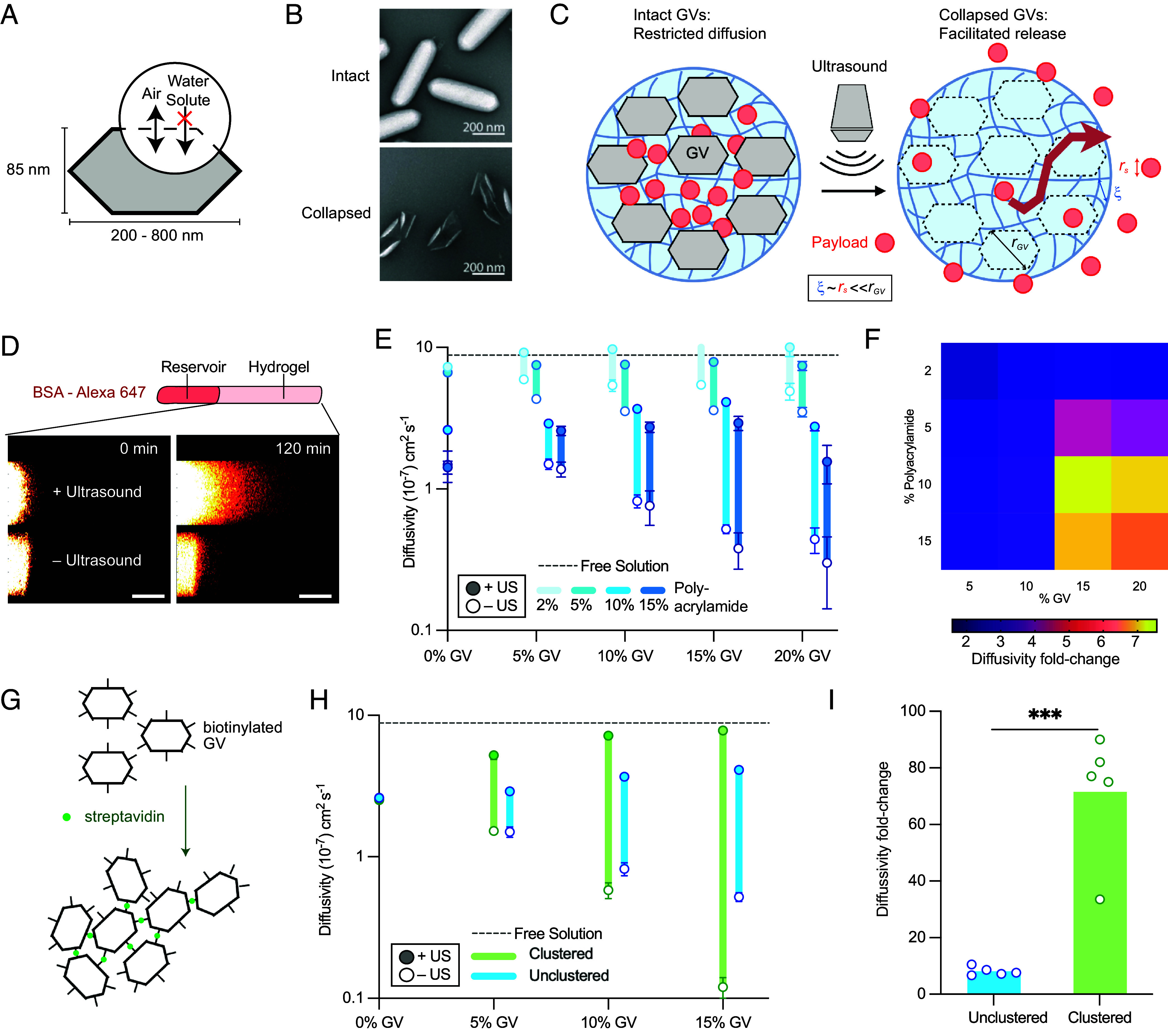
Gas vesicle (GV)–containing hydrogels produce ultrasound-modulated diffusivity changes. (*A*) Schematic of a GV highlighting its impermeability to surrounding molecules. (*B*) TEM images showing intact (*Top*) and pressure-collapsed (*Bottom*) GVs. (*C*) Diagram of the TAPS concept. Intact GVs block payload diffusion. Collapse of the GVs with a diagnostic ultrasound pulse turns them into percolation channels, allowing rapid payload release. (*D*) Illustration of the experimental setup for measuring the diffusion of BSA-AlexaFluor 647 in GV-containing hydrogels. Equation used to estimate diffusivity *D_eff_* from spatiotemporal fluorescence data *F*(*x,t*). (*E*) Diffusivity of BSA-AlexaFluor through GV gels of varying GV and polyacrylamide volume fractions with and without ultrasound exposure (*N* = 5, mean ± SEM). (*F*) Fold-change in diffusion between the intact GV “ultrasound off” and collapsed GV “ultrasound on” states for the different gel compositions in (*E*). (*G*) Schematic of GV clustering using biotin and streptavidin. (*H*) Diffusivity changes of BSA-AlexaFluor through gels containing clustered and unclustered GVs with and without ultrasound exposure for 10% polyacrylamide and varying GV volume fractions (*N* = 5, mean ± SEM). (*I*) Fold-change in diffusivity upon ultrasound exposure in 10% polyacrylamide gels containing a 15% volume fraction of clustered and unclustered GVs (*P* = 0.0002*, N* = 5).

Here, we introduce a class of hydrogel materials that respond to diagnostic ultrasound to enable targeted, image-guided drug delivery. To interact with sound waves, these materials incorporate a unique class of air-filled protein nanostructures called gas vesicles (GVs) derived from buoyant photosynthetic microbes (21, 22). Due to their ability to scatter sound waves, GVs have been developed as contrast agents for ultrasound imaging (23–28), and are smaller and more stable compared to FDA-approved microbubble contrast agents such as SonoVue and Definity. GVs comprise a 2.4 nm-thick protein shell that encloses an air-filled compartment with dimensions on the order of 100 nm that permits gas exchange between its hollow interior and the surrounding media (21, 29–31) (Fig. 1 *A* and *B*). This feature imparts GVs with indefinite stability, allowing them to remain stable in solution for several months in storage (32) and retain their structure at temperatures ranging from 25 to 60 °C (33), making them amenable for use in long-term drug formulations. In contrast, microbubbles have to be administered within hours of being activated and rapidly dissolve in circulation (34) [Sonovue half-life ~1.04 min (35)], making it challenging to perform consistent spatiotemporal localization and triggering of microbubble-derived drug carriers (36). Moreover, supporting their translational potential, GVs are nontoxic upon systemic administration (37, 38).

We hypothesized that a drug delivery approach could be implemented by taking advantage of the ability of GVs to collapse irreversibly under well-defined acoustic pressure [above a sharp threshold typically ~500 kPa (24)], causing them to lose more than 85% of their volume (29, 39) (*SI Appendix*, Table S1). We designed a hydrogel vehicle incorporating GVs alongside a protein payload, such that the presence of the drug-impermeable intact GVs sterically restricts drug diffusion, while ultrasound-triggered GV collapse instantly converts the GVs into percolation channels, leading to rapid drug release (Fig. 1*C*). Before triggering this release, the hydrogels can be imaged with lower-amplitude ultrasound to visualize their location inside the body, while GV collapse leads to a loss of ultrasound contrast (23, 40, 41) providing confirmation that release has been triggered.

In this study, we implemented this concept by generating hydrogels containing GVs and protein payloads. We performed detailed measurements of ultrasound-triggered diffusion and release, using the resulting insights to construct targeted acoustic percolation switches (TAPS). We demonstrated the ability of these devices to release proteins in the lower GI tract following oral administration and ultrasound actuation and used them to deliver an anti-inflammatory antibody drug to the duodenum in a rat model of colitis, resulting in reduced inflammation and improved health measures. TAPS enable local, image-guided, and imaging-confirmed drug delivery using a simple formulation and ubiquitously available ultrasound imaging equipment.

## Results

### GV-Containing Hydrogels Exhibit Ultrasound-Controlled Biomolecular Diffusivity.

We tested the basic hypothesis that the presence of GVs in a hydrogel would restrict the diffusion of other biomolecules by preparing polyacrylamide gels with GVs and measuring the diffusivity of fluorescently labeled bovine serum albumin (BSA). We focused on biomolecular payloads because they represent potent therapeutics that are challenging to deliver to locations such as the GI tract. We polymerized gels containing different volume fractions of GVs (purified from *Anabaena flos-aquae*) and acrylamide/bis-acrylamide inside glass capillaries and loaded an adjacent reservoir with a solution of BSA-AlexaFluor 647. Then we used confocal microscopy to track the movement of fluorescent signal through the capillary (42) (Fig. 1*D*) and fitted the resulting curves to a one-dimensional diffusion equation (*SI Appendix*, Fig. S1).

We used a diagnostic ultrasound transducer operating at 6 MHz and at short pulse durations (adapted from diagnostic ultrasound imaging) to collapse the GVs in a subset of the gels. These parameters minimize the potential for heating, cavitation, or radiation force. Our results confirmed that intact GVs restrict protein diffusion relative to GV-free hydrogels (Fig. 1*E*, empty circles), and that acoustic collapse of the GVs leads to a large increase in this diffusivity (Fig. 1*E*, filled circles). The dynamic range of diffusivity between the ultrasound “on” and “off” conditions was optimal at GV and polymer concentrations of 15% and 10% by volume, respectively (Fig. 1*F*). This is consistent with the need for low drug diffusion through the background gel, high steric blockage by intact GVs and the ability of collapsed GVs to create interconnected percolation channels. We observed no macroscopic structural or mechanical changes in the hydrogels with the addition or collapse of GVs, consistent with their relatively low volume occupancy in the gel and lack of covalent bonds to the gel network. This is consistent with our hypothesis that diffusivity switching occurs via the elimination of steric barriers upon GV collapse rather than alterations of the surrounding gel network.

The fold-change in diffusivity depends on the payload molecular weight (*SI Appendix*, Fig. S2), with smaller payloads able to diffuse more easily through the gel network around GVs.

Building on these initial results, we hypothesized that the dynamic range of our hydrogels could be widened by clustering the GVs prior to gelation. The resulting increase in inter-GV connectivity would make diffusion around intact GVs more challenging while enhancing the connectivity of the postcollapse percolation network. To test this hypothesis, we clustered GVs using biotin and streptavidin (23, 43), generating aggregates with hydrodynamic diameters of around 1 μm (Fig. 1*G*). Embedding these preclustered GVs in the hydrogel at volume fractions of 10 to 15% substantially reduced BSA diffusivity compared to unclustered GVs, while boosting the diffusivity upon GV collapse (Fig. 1*H*). The optimized composition of 15% clustered GVs and 10% acrylamide exhibited a pressure-induced diffusivity fold-change of 71.5 ± 9.8 (mean ± SEM, Fig. 1*I*).

### TAPS Enable Imaging and Spatially Targeted Drug Release In Vitro.

**Fig. 2.**
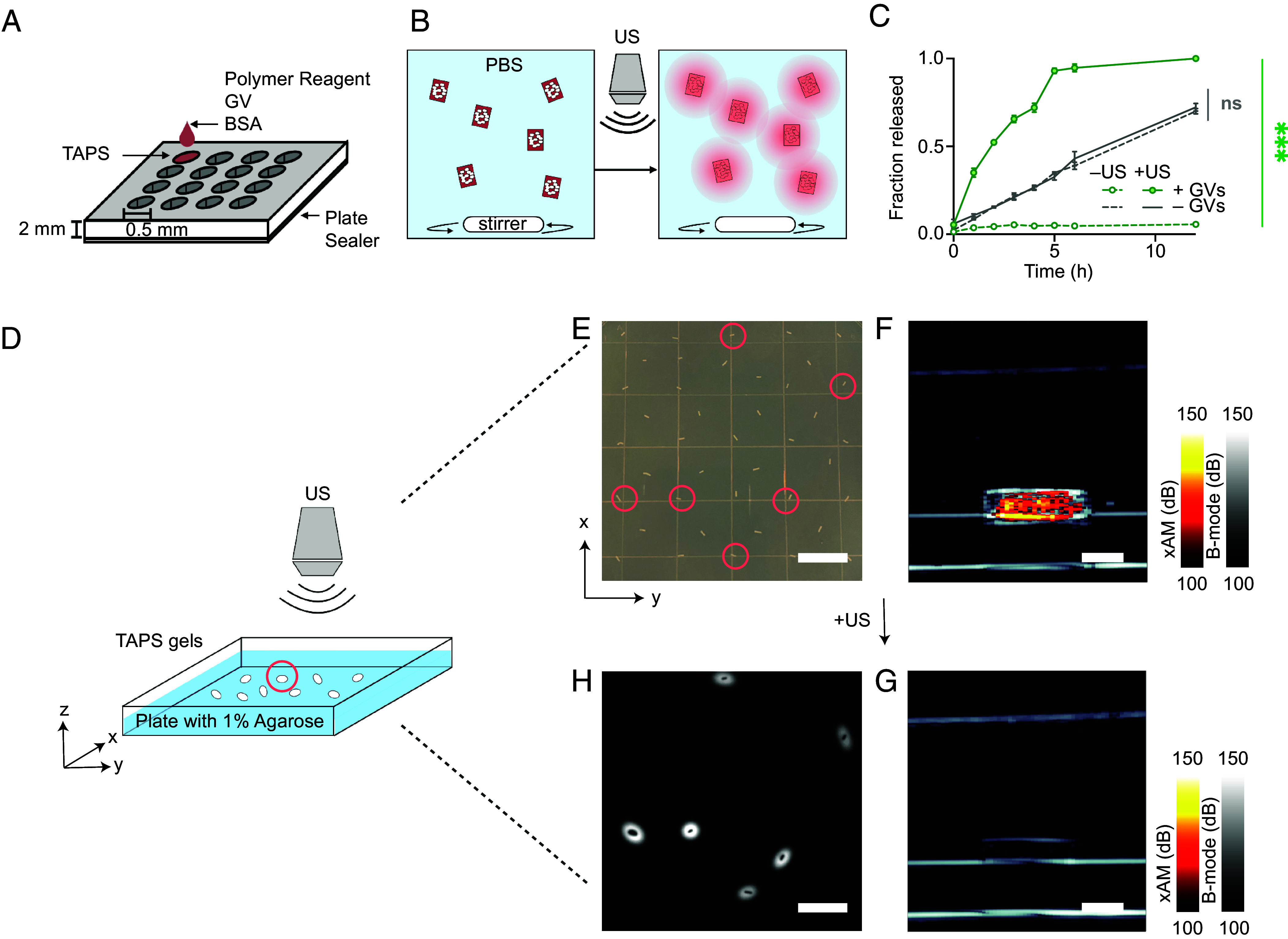
TAPS enable spatially selective ultrasound-guided, ultrasound-actuated, and ultrasound-confirmed payload release. (*A*) Schematic of the 3D-printed mold used to form millimeter-sized TAPS cylinders. (*B*) Diagram of the experimental setup used to track fluorescent payload release over time. GV gels are placed into a vial containing phosphate-buffered saline (PBS) and stirred for 12 h. The kinetics of payload release is monitored by aliquoting samples from the vial every hour and determining the fluorescence intensity. (*C*) Release profiles of clustered TAPS (green) with and without ultrasound exposure (*N* = 3, mean ± SEM, *t* test: *P* < 0.0001) compared to the gray no-GV hydrogel only controls (*N* = 3, mean ± SEM, *t* test: *P* = 0.087). Fraction released is defined as the fluorescence signal in the vessel normalized to the fluorescence signal at the end of the experiment. (*D*) Diagram of the experimental setup used to assess spatial targeting performance of the drug delivery system in vitro. TAPS are dispersed on a 1%wt agarose phantom, and an ultrasound probe is used to target individual gels for collapse. (*E*) *Top–Down* photo of the plate showing dispersed TAPS in agarose. Devices circled in red depict those selected for ultrasound targeting at t = 0. (*F*) xAM ultrasound image of a representative TAPS prior to ultrasound actuation. (*G*) xAM image of the same TAPS after ultrasound activation. (*H*) Fluorescence subtraction image obtained between t = 12 h post–GV collapse and at t = 0. (Scale bar, *E* and *H*, 10 mm; *F* and *G*, 1 mm.)

Having demonstrated switchable diffusivity, we set out to test the ability of GV-containing gels to release drug payloads on command. We formed prototype TAPS devices by mixing hydrogel-forming reagents, clustered GV and fluorescent BSA in 3D-printed cylindrical molds with a diameter of 0.5 mm and a height of 2 mm (Fig. 2*A*). The gels were then preincubated in a phosphate-buffered saline (PBS) solution for 2 h to remove loosely bound BSA on or near the hydrogel surface and transferred into a stirred vial containing fresh media (Fig. 2*B*). We applied ultrasound to a subset of the gels and tracked the fluorescence intensity of the solution over 12 h, finding that while TAPS gels retained most of their payload in the absence of ultrasound treatment, devices actuated by ultrasound released most of their BSA within 2 h and reached nearly complete release in 5 h (Fig. 2*C*).

Having demonstrated payload release from these model TAPS, we tested their ability to be imaged with ultrasound and actuated in a spatially selective manner. We dispersed our devices in 1% agarose with interdevice spacing of approximately 5 mm (Fig. 2*D*). We used a diagnostic ultrasound transducer operating at 6 MHz and 200 kPa peak positive pressure to locate and image the gels using a GV-selective nonlinear xAM pulse sequence (44) (Fig. 2*F*). The gels were readily visible under ultrasound. We then selected several individual TAPS gels (indicated by the dashed red circles in Fig. 2*E*) for actuation by increasing the ultrasound transmit pressure to >550 kPa, leading to GV collapse, as confirmed immediately by the disappearance of their xAM ultrasound contrast (Fig. 2*G*). We observed that this remote actuation of the gels resulted in selective payload release, as shown by the localized diffusion of fluorescent BSA into surrounding agarose (Fig. 2*H*). These results validate the fundamental capability of TAPS to be used as ultrasound-imaged, ultrasound-actuated, and ultrasound-confirmed delivery vehicles.

### TAPS Enable Spatially Selective Ultrasound-Triggered Drug Release In Vivo.

**Fig. 3.**
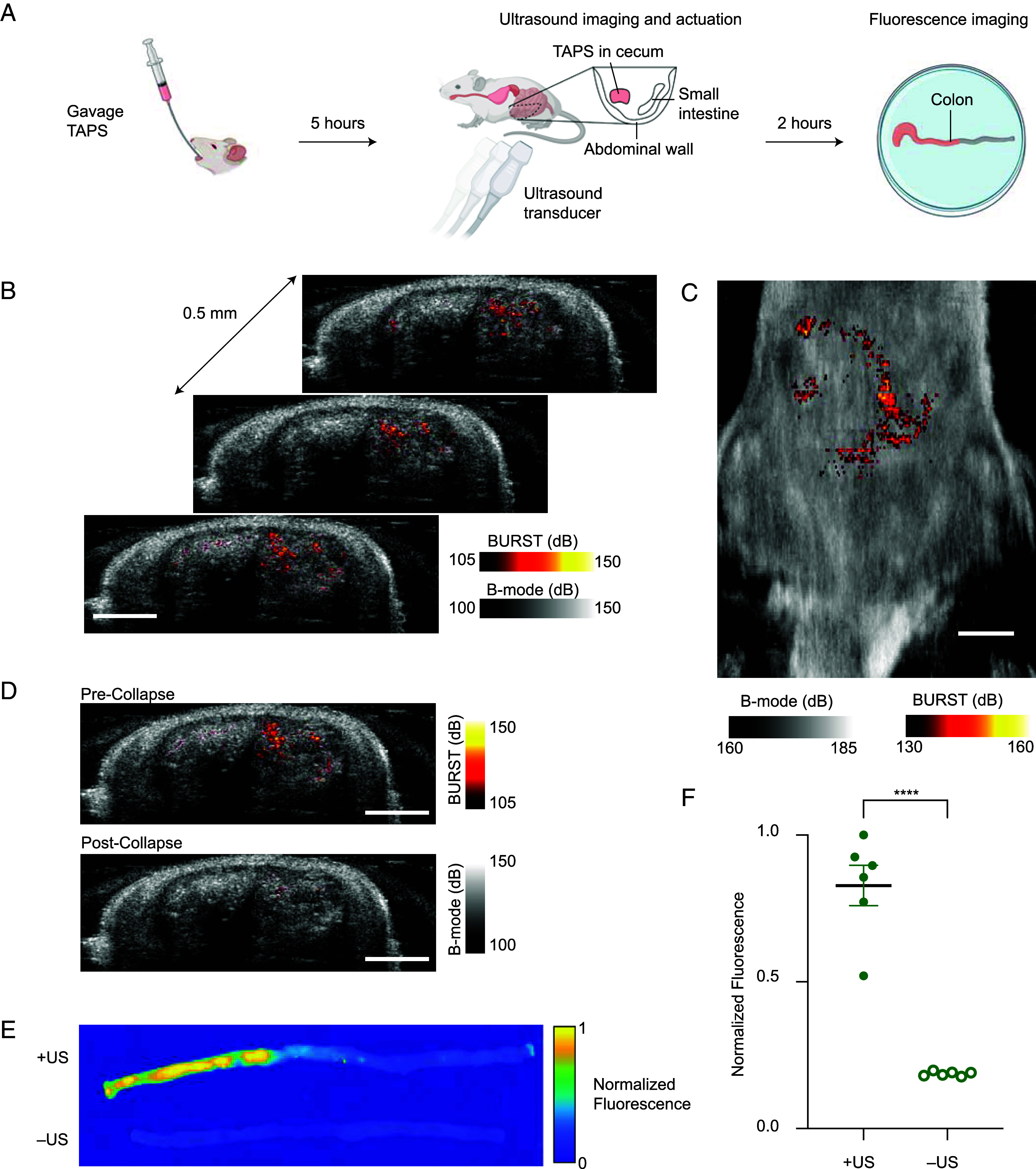
Ultrasound imaging and targeted payload release from TAPS in the gastrointestinal (GI) tract. (*A*) Illustration of the in vivo imaging and release experiment. (*B*) Representative stack of coronal BURST ultrasound images (heat color map) of the lower abdomen area of a rat overlaid on anatomical B-mode images (grayscale) five hours after TAPS gavage. BURST signal was not observed in rats that were given saline (*SI Appendix*, Fig. S3). (*C*) Representative *Top–Down* projection of BURST and B-mode signal across all the imaged planes. (*D*) Representative ultrasound images of the cecum preactuation (*Top*) and postactuation of release from the TAPS gels. (*E*) Representative fluorescence images of colons from rats gavaged with TAPS, with and without ultrasound activation of payload release. (*F*) Retained fluorescence signal in the colons of TAPS-gavaged rats with and without ultrasound actuation (*N* = 6, mean ± SEM, *t* test: *P* < 0.0001). (Scale bar, *B*–*D*, 1 cm.)

One of the most immediate applications of spatially triggered drug delivery devices is the GI tract, due to a range of GI pathologies requiring local treatment (45). Thus, we chose the GI tract as the initial proving ground for TAPS. To demonstrate the spatiotemporal control of drug release in vivo, we used gavage to orally administer a suspension of BSA-loaded TAPS in PBS to Sprague Dawley rats (Fig. 3*A*). After 5 h, we used a diagnostic ultrasound probe transmitting at 11.4 MHz to locate GV gels in the ileum and cecum (Fig. 3 *B* and *C* and *SI Appendix*, Fig. S3), using GV-selective BURST mode for maximum detection sensitivity (40, 46–48) in the GI tract with its relatively complex and heterogeneous tissue and contents. Depending on the location of the target organ, other ultrasound modes capable of both 2D imaging and triggering GV collapse such as B-mode and nonlinear imaging (44, 49) may be more convenient and could be considered for different applications. We then triggered payload release by increasing the transmit pressure to >550 kPa and confirmed GV collapse by the disappearance of BURST signal (Fig. 3*D*). We then collected the large intestines of the rats and laid them out in PBS. Following established protocols (50, 51), we gently flushed the intestines with PBS after 12 h to evacuate TAPS and any solid debris, leaving behind fluorescently labeled protein that was released from the gels and absorbed by intestinal walls. Fluorescence images of the GI tract showed significant signal localized in the proximal colon only in rats that received the release-activating ultrasound pulse (Fig. 3 *E* and *F*). These results show that TAPS can be imaged and triggered noninvasively with ultrasound inside intact living animals, leading to localized release of a protein payload.

### TAPS Release of Etanercept in the GI Tract Enables the Treatment of Colitis.

Having established the basic functionality of TAPS in vitro and in vivo, we set out to demonstrate their application to delivering a protein therapy in a preclinical disease model. Inflammatory bowel diseases, including ulcerative colitis, affect more than 2 million people in the United States (52). Patients with colitis often require lifelong treatment and pain management, experiencing reduced quality of life due to episodes of relapse characterized by moderate to severe lesions in the large intestine. Among other treatments, colitis is treated with biologics inhibiting the proinflammatory cytokine tumor necrosis factor (TNF) (53). These drugs are administered systemically, typically requiring high systemic doses to reach therapeutic concentrations at the sites of GI inflammation. The resulting systemic immunosuppression can lead to side effects such as infections and tumorigenesis (54–56). If anti-TNF therapies could be delivered more directly to the colon, this would increase their dose at the target site while mitigating systemic side effects. We hypothesized that TAPS would allow us to deliver the monoclonal anti-TNFα antibody etanercept to the colon and thereby treat colitis in a rat model of the disease. Etanercept is FDA approved for the treatment of autoimmune diseases and has previously been validated to treat experimental colitis in rodents due to its cross-reactivity with rat TNFα (57–59).

**Fig. 4.**
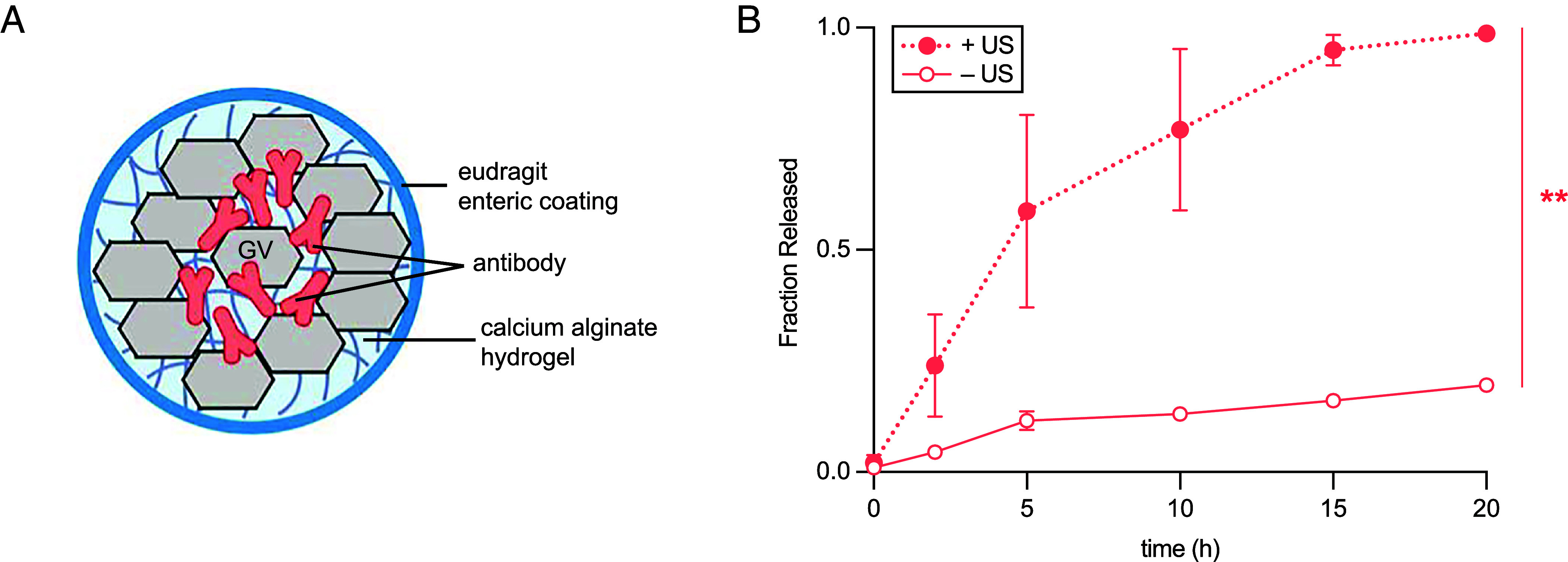
TAPS design for oral delivery of biomolecules. (*A*) Illustration of antibody-loaded TAPS based on calcium alginate. TAPS for oral gavage in rodents are coated with a thin enteric coating layer of Eudragit. (*B*) Ultrasound-mediated controlled release kinetics of BSA-AlexaFluor 647 from calcium alginate-based TAPS in simulated gastric fluid (*N* = 3, mean ± SEM, *t* test: *P* < 0.002).

To formulate TAPS with etanercept, we incorporated the drug and GVs in calcium alginate – a hydrogel validated for sustained release of monoclonal antibodies (60). To protect the hydrogels from stomach pH and enzymes, we dip-coated them with Eudragit FL30D55 (Fig. 4*A*). The ultrasound-mediated protein release performance of this system was validated by releasing fluorescent BSA in simulated rat gastric fluid to simulate the intended in vivo environment (Fig. 4*B*).

**Fig. 5.**
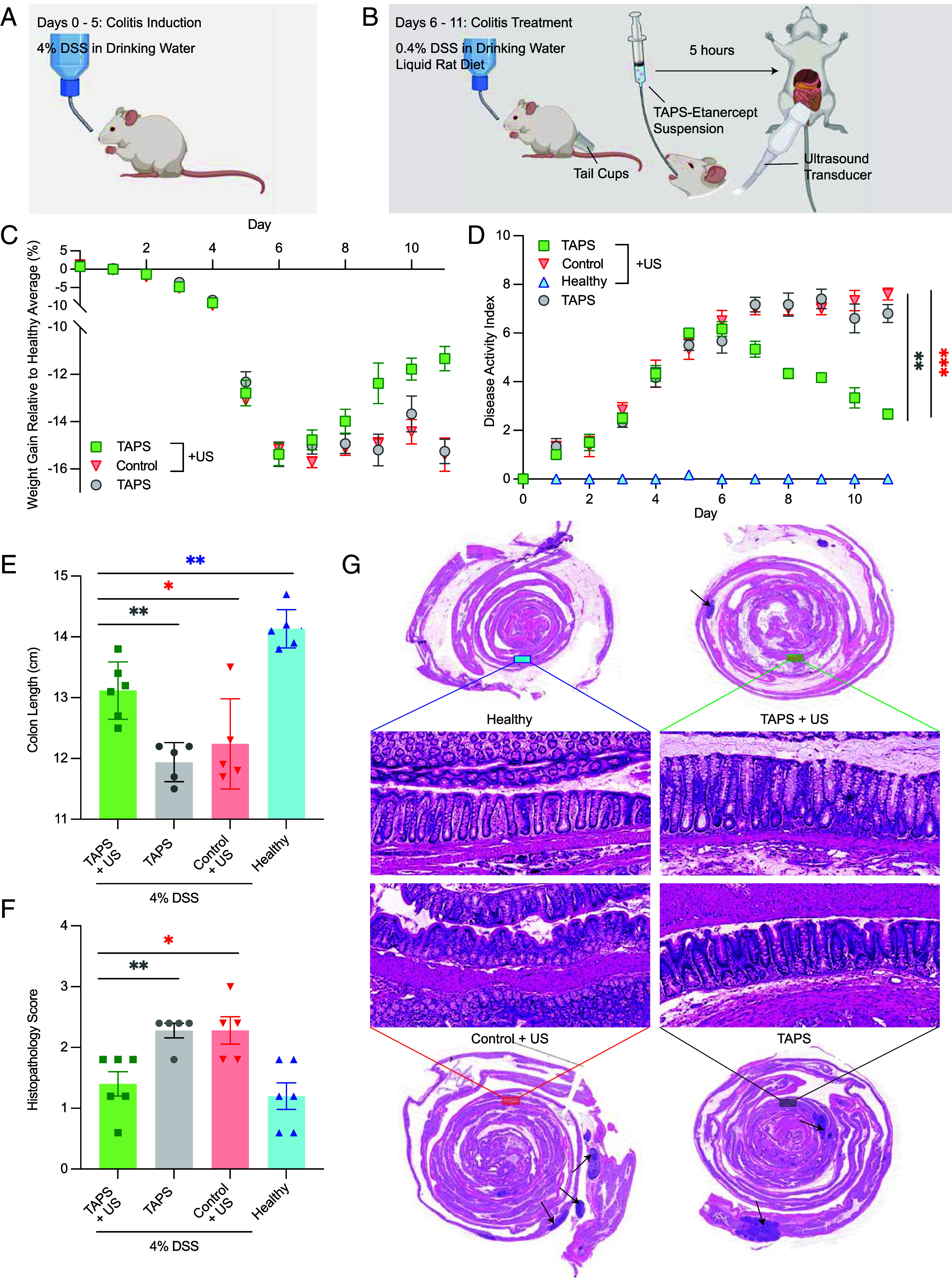
TAPS with etanercept enable ultrasound-triggered treatment of colitis in a rat model. (*A* and *B*) Experimental workflow used to demonstrate treatment of colitis in rats (*N* = 6, mean ± SEM) using the TAPS delivery vehicles. (*C*) Change in rat weight gain rate relative to healthy controls over the treatment period for rats with severe colitis that were given TAPS treatment or controls. Where not seen, error bars are smaller than the symbol. The *t* test *P*-values for the statistically significant points during the treatment period are as follows for i) TAPS+US vs TAPS: Day 9, *P* = 0.0343; Day 11, *P* = 0.0004 ii) TAPS+US vs control: Day 9, *P* = 0.01783; Day 10, *P* = 0.0035; Day 11, *P* = 0.0010. (*D*) Time course of the Disease Activity Index (DAI), which is the sum of the stool consistency index (0 to 3), fecal bleeding index (0 to 3), and weight loss index (0 to 4). The *t* test *P*-values for the statistically significant points during the treatment period are as follows for i) TAPS+US vs TAPS: Day 7, *P* = 0.0023; Day 8, *P* = 0.0003; Day 9, *P* = 0.00002; Day 10, *P* = 0.0013; Day 11, *P* < 0.00001 ii) TAPS+US vs control: Day 7, *P* = 0.0027; Day 8, *P* = 0.00001; Day 9, *P* < 0.00001; Day 10, *P* = 0.000053; Day 11, *P* < 0.00001. (*E*) Colon length of rats after the indicated treatments. The *t* test *P*-values are (vs TAPS+US) i) TAPS: *P* = 0.0011 ii) control: *P* = 0.0405 iii) healthy: *P* = 0.0013. (*F*) Histopathology scores for rats in (*B*) and a no-DSS health control group. The *t* test *P*-values are (vs TAPS+US) i) TAPS: *P* = 0.0060 ii) control: *P* = 0.0166. (*G*) Representative H&E stains of the colonic sections of different rat groups after the indicated treatment. Black arrows are pathologist-identified abscessed regions.

We induced severe colitis in Sprague-Dawley rats by adding 4 percent dextran sulfate sodium (DSS) to their drinking water for five days (Fig. 5*A*), confirming that DSS-treated rats lost weight relative to untreated controls (*SI Appendix*, Fig. S4). Starting day 6, we treated the animals with daily oral gavage of TAPS-etanercept at a dose of 83 µg/g-rat, which was not sufficient in the format of a free antibody solution to reverse weight loss (*SI Appendix*, Fig. S5). We hypothesized that with TAPS encapsulation and ultrasonic release in the GI tract, this low dose could be sufficient for weight recovery and amelioration of other disease markers.

In a subset of subjects, at 5 h after gavage, when the gels are expected to be in the cecum (Fig. 4 *B* and C), we triggered etanercept release using a diagnostic ultrasound transducer scanned over the abdomen with transmit pressure >550 kPa (Fig. 5*B*). Control conditions included rats that were administered TAPS-etanercept but not ultrasound and rats that were treated with ultrasound but no TAPS. Before and during the treatment window we measured the rats’ weight and disease activity index (DAI) (61), an aggregate measure encompassing stool consistency, fecal bleeding, and weight loss. The subjects were euthanized at day 12 for blinded histopathological analysis of the colon by a nonaffiliated pathologist.

In rats that received ultrasound-actuated TAPS-etanercept treatment, we observed a significant recovery of body weight (Fig. 5*C*), reversal of the DAI (Fig. 5*D*), recovery of colon length (Fig. 5*E*) and reduced colon tissue damage (Fig. 5 *F* and *G*) compared to controls. Postmortem histological imaging revealed that the treated group had mild to no histological alterations by the end of the treatment, with regular structure of the lamina propria restored and comparable to those in the healthy rat controls. This evaluation (*SI Appendix*, Tables S2 and S3) also did not find any evidence of mechanical damage resulting from ultrasound exposure such as necrosis and blood extravasation (62), consistent with our use of high frequencies and short pulse durations well outside the parameter ranges associated with cavitation or heating. In contrast, rats that were administered TAPS without ultrasound actuation or ultrasound alone had crypt abnormalities and inflammatory acute infiltration of the subepithelium and lamina propria (Fig. 5*G*).

Together, these results demonstrate the capability of TAPS to deliver an efficacious biologic disease treatment to the GI tract following oral administration.

## Discussion

This work establishes TAPS as a unique drug delivery system allowing everyday diagnostic ultrasound devices to image, trigger, and confirm the release of therapeutics at specific locations inside the body. TAPS leverages the ability of GVs to simultaneously act as ultrasound contrast agents and collapsible steric blockers, allowing simple hydrogels to be both visualized and remotely actuated with ultrasound.

In this study, we established the fundamental concept of a hydrogel in which drug diffusion is activated by ultrasound, demonstrated the ability of this concept to support remote-controlled release of biomolecular payloads, and provided a proof of concept for its use to deliver a biologic therapy to the GI tract. In future studies, the TAPS technology can be extended to a wider range of cargoes (63)—from small-molecule drugs (~1 nm) to viruses such as adeno-associated virus (>20 nm)—by tuning the hydrogel mesh size relative to the hydrodynamic radius of the payload. These payloads could be released in contexts ranging from ingestible pills to implantable and interventional medical devices or smart robots navigating through a tissue (64). In addition, a combination of GV types with varying collapse pressures (24) could be used to sequentially release multiple payloads. Furthermore, acoustophoresis could be used to pattern GVs within the material (65) to further optimize release properties.

As with any new technology, further work is needed to optimize the TAPS system and enable its broadest range of clinical applications. Depending on the therapeutic payload, it may be necessary to use hydrogels other than the polyacrylamide and alginate used in this study and establish their compatibility with GVs. Additionally, for repeated administration of GV-containing hydrogels, it will be important to characterize the immune accessibility and potential immunogenicity of the GV proteins. Furthermore, for patients to be able to use TAPS at home, consideration must be given to ultrasound devices, including low-cost portable devices (66), adhesive ultrasound (67–70) and machine learning algorithms to automatically recognize and trigger delivery vehicles at specific anatomical sites. For uses in the GI tract, it may also be necessary to deal with confounding gas and solid contents in ultrasound images. This task will be aided by GV-specific ultrasound pulse sequences (40, 44, 49, 71) and patient preparation through diet.

Notwithstanding the need for further research, the relative simplicity and versatility of the TAPS approach and its compatibility with existing FDA-approved hydrogel materials, drugs, and ultrasound devices will allow this technology to percolate toward valuable clinical applications.

## Materials and Methods

### Animals.

5-week-old male Sprague Dawley rats weighing between 150 to 160 g were purchased from Charles River. The rats were housed in the animal facility of the Office of Laboratory Animal Resources (OLAR) at the California Institute of Technology with a 13 h/11 h light/dark cycle with cage conditions kept between 22 to 24 °C and 30 to 70% humidity. The rats were monitored daily for overall well-being and given ad libitum access to water and standard laboratory chow. All animals were acclimated for three days prior to performing experiments that are in accordance with an approved Caltech Institutional Animal Care and Use Committee (IACUC) protocol, including humane euthanasia.

### Preparation of GVs.

*Anabaena flos-aquae* cultures were grown for several weeks under controlled gaseous conditions with appropriate illumination. Once cultures reached sufficient density, buoyant GV-producing cells were separated using a separatory funnel and subsequently lysed through hyperosmotic shock, from which GVs were purified via centrifugally assisted floatation following established protocols (23, 39). Purified GVs were resuspended in PBS and their concentration was determined by measuring optical density at 500 nm (OD_500_). GVs underwent modification through removal of GvpC (AnaΔC) as previously described and biotinylation was performed using sulfo-NHS-LC-biotin followed by incubation with streptavidin for clustering experiments to induce aggregation.

### Diffusion Characterization.

50 μl of hydrogel reaction mixture is made by copolymerizing a predetermined volume fraction of acrylamide and bis-acrylamide (A7168: Sigma-Aldrich) in prefiltered pH 7.4 PBS solution at a fixed cross-linking density of 2.7%. 50 μl of purified *Anabaena Flos-Aquae* GVs solution was concentrated to a predetermined volume fraction (optical density, OD 24 = 1% vol. GV) and added to the hydrogel reaction mixture. The hydrogel reaction mixture is then degassed in a vacuum chamber for 30 min to rid the system of oxygen. 0.1 μl and 0.5 μl of initiators tetramethyl ethylene diamine (T9281: Sigma-Aldrich) and 10% ammonium persulfate solution (A3678: Sigma-Aldrich), respectively, were then added to the reaction mixture and agitated slightly using a pipette to start the free-radical polymerization at room temperature (25 °C). Using the prepared syringe-capillary system, the hydrogel reaction mixture is immediately withdrawn into the glass capillary, leaving about a 1 inch of the glass capillary empty at the top for dye loading later. This recipe is expected to produce hydrogels of approximately 19 nm (72) pore sizes. The bottom of the glass capillary is quickly capped with some clay to prevent the hydrogel from drying or leaking out. To collapse the GVs in the GV gel, the filled glass capillary is first placed into a PBS-filled container and held in place by solidified 1% Agarose. An L10-4 V 128-element linear array ultrasound transducer (Verasonics) operating at peak positive pressure of 600 kPa was then held in place over the capillary for 3 mins to collapse the GVs in the gel. GV collapse is verified visually from the loss of gel opacity, and from the loss of ultrasound signal within the gel. The capillary-loaded gels are then equilibrated at room temperature with PBS for at least 6 h prior to experiments.

Albumin from Bovine Serum (BSA) with AlexaFluor™ 647 conjugate (A34785: ThermoFisher Scientific) was diluted to 500 μg/ml using PBS and equilibrated at room temperature for 5 mins. A 1 ml BD Luer-Lok™ tip syringe fitted with a blunt 30G needle (SAI Infusion Technologies) was then used to inject the protein dye near the interface of the hydrogel casted within a 3-inch open-ended glass capillary (1B120-3: World Precision Instruments), ensuring all the air bubbles are extracted prior to experiments. Both ends of the capillary are then capped with clay to prevent evaporation. The experiments were then carried out as described (42) for rapid measurements of protein diffusion through hydrogels using a Zeiss LSM 800 inverted microscope fitted to a 2.5× objective lens (numerical aperture: 0.075). Fluorescence imaging was performed under an Alexa 647 channel (excitation: 587/25 nm, emission: 647/70 nm) and images were taken every 6 mins over a period of 2 h.

The time series of fluorescence images from the capillary diffusivity experiments were imported into MATLAB for image processing. The time evolution of the fluorescence intensity across the capillary length from the fluid–gel interface was recorded and averaged across the capillary cross-sectional area. Since the length of the gel (5 cm) is significantly larger that its diameter (0.6 mm) one can approximate the protein-dye transport in the gel to follow a 1-D diffusion model through an infinite slab given by the following equation:

[1] $$F\left(x,t\right)=K\;erfc\left(\frac{x}{2\sqrt{D_{\mathrm{eff}}\;t}}\right),$$

where *K* and *D_eff_* are constants that represent the gel partitioning and diffusion coefficients of BSA, respectively, *F* is the fluorescence intensity normalized to the dye reservoir intensity, *x* is the distance from the interface, and *t* is the time. The experimental data were then fitted to Eq. **1** using a nonlinear programming solver on MATLAB to obtain the two constants.

### Acrylamide TAPS Preparation.

The acrylamide TAPS cargoes utilized the same hydrogel recipe as described in the BSA diffusivity experiments but with the addition of BSA-AlexaFluor 647 at a final dye concentration of 500 μg/ml also mixed into the polymerization precursor media. The reaction mixture was then pipetted into 0.4 μl cylindrical molds and allowed to polymerize completely overnight in a humidified chamber to prevent drying out. Prior to the experiment, the gels are incubated in PBS for 2 h to remove loosely bound proteins. Collapse of GVs in the gel utilize the protocol described previously for diffusion characterization.

### Calcium Alginate TAPS Preparation.

Hydrogels were formed using solutions of 2 wt.% alginate (102877-746: VWR) in PBS solutions containing 15 vol.% clustered GVs, 25 mg/mL of etanercept (Y0001969: Sigma-Aldrich) reconstituted in bacteriostatic water as per the supplier’s guidelines, calcium carbonate (CaCO_3_) (239216: Sigma-Aldrich) and D-Glucono-δ-lactone (GDL) (8.43794: Sigma-Aldrich). To make a homogenous gel, CaCO_3_ was added to 2 mL of alginate solution to obtain a molarity of 144 mM and vortexed for 10 s and then left to degas at 37 °C overnight. 100 μL of the etanercept solution was then added to the mixture followed by GDL till it doubled the molarity of CaCO_3_ to maintain a neutral pH. The resultant solution is gently mixed to prevent bubbles from being reintroduced and pipetted into 0.4 μl cylindrical molds and allowed to polymerize completely overnight in a sealed humidified container within an incubator at 25 °C to prevent it from drying out and keep the hydrogels robust. Calcium alginate gels prepared using this method are expected to possess pore sizes of around 5 nm (73). They are then incubated in PBS for 2 h to remove loosely bound proteins. The gels were then enterically coated by lightly dip coating them in organic solutions of Eudragit FL30D55 (Evonik Industries) in acetone containing 3 vol.% ethanol and gently blasted with air to dry. The gels were then suspended in PBS prior to administration. This recipe makes enough therapeutic for 2 rats/day at a dose of around 12.5 mg/rat-day. To prepare for oral gavage, a 3 mL syringe was backfilled with 1 mL of PBS. Using a second syringe, TAPS are slowly withdrawn into the gavage needle. The gavage needle is then connected back to the first syringe.

### In Vitro Release.

100 pieces of the acrylamide TAPS were suspended in 30 ml fresh PBS and stirred at 37 °C and 100 rpm. The experiment was performed in a dark room to minimize fluorophore photobleaching. Every 2 h for a total of 12 h, 10 μL of the suspension was aliquoted into a 90-well plate containing 100 μL of PBS. The wells were then analyzed using a Spectramax M5 plate reader with the following wavelength settings: excitation: 587/25 nm and emission: 647/70 nm. After 72 h, the suspension was aliquoted again to determine the total protein-dye payload present in all ten TAPS cargoes. Fluorescence intensities collected during the time series were then normalized to the final intensity at 72 h to determine the release kinetics of the TAPS cargoes.

### In Vitro Spatial Targeting.

Acrylamide TAPS were evenly distributed and spaced at around 1 mm in a shallow petri dish filled with 1% agarose and allowed to set. An L10-4 V 128-element linear array ultrasound transducer (Verasonics) was then operated at 6 MHz at peak positive pressure of 200 kPa and low duty cycle (<<1%) to image the gels, and subsequently at approximately 600 kPa to trigger the release of fluorophores into the agarose for preselected TAPS. We do not expect the choice of transducer frequency and pulse duration to affect GV collapse efficiency and imaging sensitivity as shown in recent studies (23, 39, 74, 75).

### In Vivo Spatial Targeting and Release.

Rats were fasted and given sucrose water, their abdomen hair shaved, and tail cups were placed 17 h prior to experiments to minimize coprophagy. 1 mL acrylamide TAPS suspension was orally gavaged into the rats. BURST imaging was then performed on the entirety of the rat abdomen 5 h postgavage to verify arrival of the GV gel bolus in the cecum and subsequently trigger the release of fluorophores. BURST images were acquired using a Verasonics Vantage programmable ultrasound system with an L10-4 V 128-element linear array ultrasound transducer attached to a custom-made motor stage to scan across the entire abdominal area of the rats. The motor stage was programmed to move the transducer in 0.5 mm steps between transverse planes starting at the bottom of the rib cage and moving toward the tail (approximately 120 to 160 per rat) and to acquire 2 side-by-side transverse planes to capture the entire width of the abdominal area. To mitigate issues due to tissue motion, images were acquired using a rapid BURST script as described previously (40) that transmits and acquires three 32-aperature focused beams at a time to improve the frame rate by a factor of 3. The transmit waveform frequency was set to 11.4 MHz to improve the spatial resolution, the focus was set to 12 mm centered on the approximate depth of the cecum, and the number of half-cycles was set to three to increase the BURST signal. BURST images were generated using the temporal-template unmixing algorithm as described previously (40) on the first three frames which consisted of a precollapse frame acquired at 1.6 V and two collapsing frames acquired at 30 V. ROIs were manually drawn around the intestines, and BURST acquisitions that occurred during breathing were automatically excluded if the total BURST signal outside the ROI exceeded 162 dB. For display, the BURST signal was summed over the depth of the ROI, excluding pixels below 1 × 10^5^ to reduce background, and the B-mode signal was summed from a depth of 5 to 18 mm. After imaging, the animals were then euthanized, and the colon was harvested and analyzed for fluorescence under a ChemiDoc.

### DSS-Induced Ulcerative Colitis Model.

Severe ulcerative colitis was induced in rats allowing rats to consume 4% by weight of dextran sulfate sodium (36 to 50 kDa colitis-grade DSS, MP Biomedicals) in their drinking water for five days. On day 6, DSS is reduced to 0.4% and TAPS treatment is given once a day for six days until euthanized on day 11. During these 11 d, the rats were monitored daily for changes in weight and stool quality.

### TAPS Treatment.

During TAPS treatment, the standard laboratory chow is replaced with rodent liquid diet (AIN-76, Bio-Serv) and custom fit tail cups are attached on each rat to prevent coprophagy to improve ultrasound imaging performance. Calcium alginate TAPS loaded with etanercept as previously described were utilized in the TAPS treatment and are freshly prepared prior to daily treatment. It is worth noting that etanercept or human Enbrel is not an FDA-approved treatment for colitis, but rather for rheumatoid arthritis (76). However, we used it as it is a common human anti-TNF-α prescription with significant binding to rodent TNF-α without requiring a surrogate, unlike infliximab (77, 78). 1 mL of the hydrogel suspension was orally gavaged into the rats. 5 h after administration, the rats are anesthetized and placed on a holder containing an ultrasound transparent window to enable imaging, verify TAPS arrival in the cecum, and trigger GV collapse for etanercept release in the colon using the same imaging parameters described above except a collapsing voltage of 50 was used for 18 frames and a single focused beam was acquired at a time to maximize collapse throughout the GI tract.

### DAI.

The DAI was scored using the following criteria: stool consistency (hard: 0, soft: 2, and diarrhea: 4), fecal occult blood using Hemoccult Sensa (Beckman Coulter) (negative: 0, positive: 2, and macroscopic: 4), and decrease in weight relative to the average weight of the healthy controls (less than 1%: 0, 1 to 5%: 1, 5 to 10%: 2, 10 to 20%: 3, and more than 20%: 4).

### Histopathology and Scoring.

Tissue samples of the colon were fixed in 3.7% paraformaldehyde overnight at 4 °C, dehydrated in ethanol, and embedded in paraffin. Hematoxylin and eosin (H&E) staining was then performed on the tissues for general histological observation. The histological scores were assessed in a blinded manner for inflammation severity (none: 0, slight: 1, moderate: 2, and severe: 3), polymorphonuclear neutrophil (PMN) infiltration/high power field (HPF) (less than 5: 0, 5 to 20: 1, 21 to 60: 2, 61 to 100: 3, and more than 100: 4), injury depth (none: 0, mucosa: 1, submucosa and mucosa: 2, and transmural: 3), crypt damage (none: 0, basal 1/3: 1, basal 2/3: 2, only surface epithelium intact: 3, and total crypt lost: 4). The final score was then averaged across all indicators to determine disease severity.

## Supplementary Material

Appendix 01 (PDF)

## Data Availability

All study data are included in the article and/or *SI Appendix*.
