# Supplementary material for: Acoustic percolation switches enable targeted drug delivery controlled by diagnostic ultrasound
Source: Proc Natl Acad Sci U S A. 2025 May 14;122(20):e2423078122. doi: 10.1073/pnas.2423078122 (PMC12107142; doi:10.1073/pnas.2423078122)
Supplement: Supplementary file 1 — Appendix 01 (PDF) [file pnas.2423078122.sapp.pdf]

SUPPLEMENTARY INFORMATION

Supplementary Table 1 – GV dimensions (mean ± s.e.m.) measured using EM for use in volume estimations[23,24]. The GV shell thickness is estimated to be 2.4 nm. Intact GVs are approximated as cylindrical in shape. Collapsed GVs are assumed to be flattened, leaving just the shell.

Anabaena flos-aquae GVs	
Length (nm)	519 ± 160
Width (nm)	85 ± 4
Total volume (nm^3)	2.95E6
Shell volume (nm^3)	3.60E5
Estimated volume ratio (collapsed / intact)	12.2%

Supplementary Figure 1 – Representative raw data for diffusivity measurements. a, Representative fluorescence images at two time points (top) and quantified normalized fluorescence profiles in the capillary diffusion experiment. **b,** Error function used to obtain diffusion coefficients.

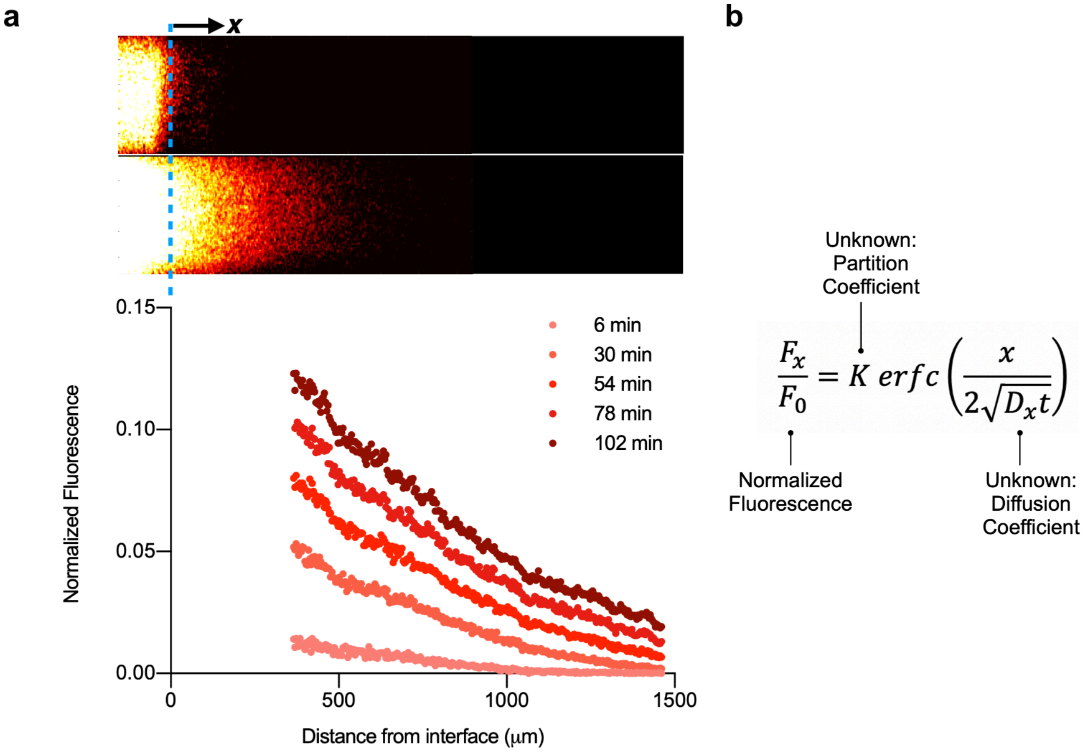

Supplementary Figure 2 – Effect of Molecular Weight on Ultrasound-Triggered Diffusivity Change. Effectiveness of gas vesicle-containing (15% vol.) polyacrylamide hydrogels (10% vol.) as ultrasound triggered delivery vehicles as a function of payload molecular weight **a,** Diffusivity of unconjugated AlexaFluor (1.3 kDa), BSA-AlexaFluor (~68 kDa) and AlexaFluor conjugated Etanercept (~150 kDa) with and without ultrasound exposure ($N = 5$, *mean* ± *s.e.m.*) **b**, Fold-change in diffusivity upon ultrasound exposure of gels described in **(a)**.

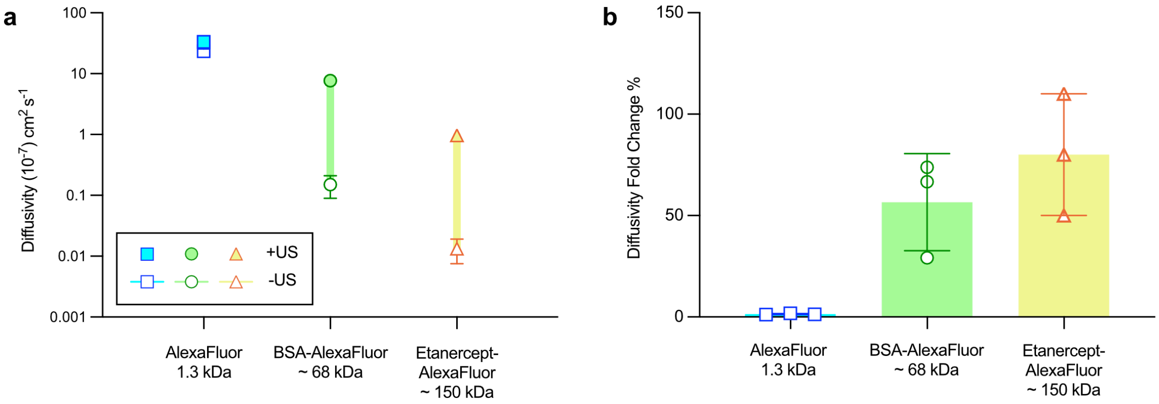

Supplementary Figure 3 – Additional images of TAPS-gavaged and control animals. a-b, Additional BURST images of **(a)** rats gavaged with TAPS as described in Fig. 3c and **(b)** controls that did not receive TAPS. **c,** Individual measurements, mean and SEM of total abdominal BURST signal in rats receiving TAPS or controls. Scale bars represent 1 cm.

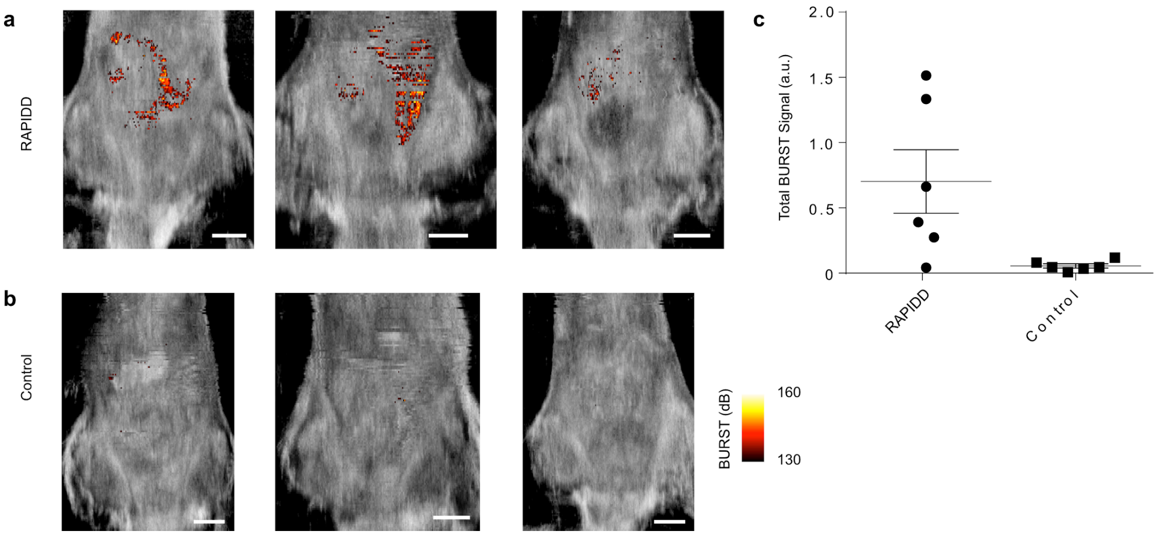

Supplementary Figure 4 - Weight gain trends for healthy and DSS-treated rats. Average weight of rats receiving the DSS treatment described in Fig. 5a or untreated controls.

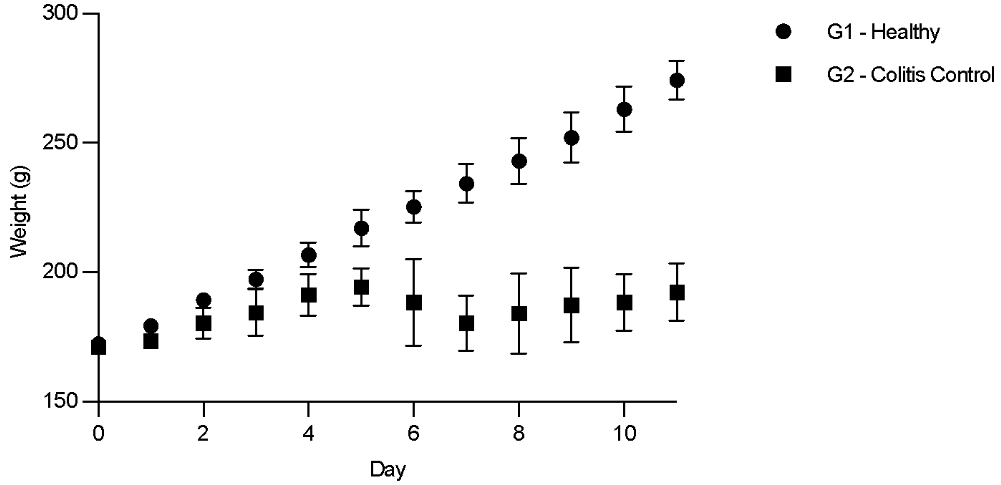

Supplementary Figure 5 – Dosing experiment of TAPS-free etanercept administration in a rat colitis model. Change in weight gain of rats administered oral gavage of Etanercept (circle markers) of concentrations 83, 333 and 667 µg/g-rat daily from treatment days 6 to 11. At an etanercept concentration of 83 µg/g-rat, the weight of the rat stagnates during the treatment phase indicating low antibody uptake. In contrast, we see those rats given TAPS + ultrasound treatment (square markers) achieve weight recovery not previously observed at this concentration during oral gavage (*N* = 5, *mean* ± *s.e.m*, t-test: *P* = 0.0052). This recovery attained by day 11 is comparable to the oral gavage of neat 333 µg/g-rat etanercept at a quarter of the dosage when encapsulated in our delivery vehicle.

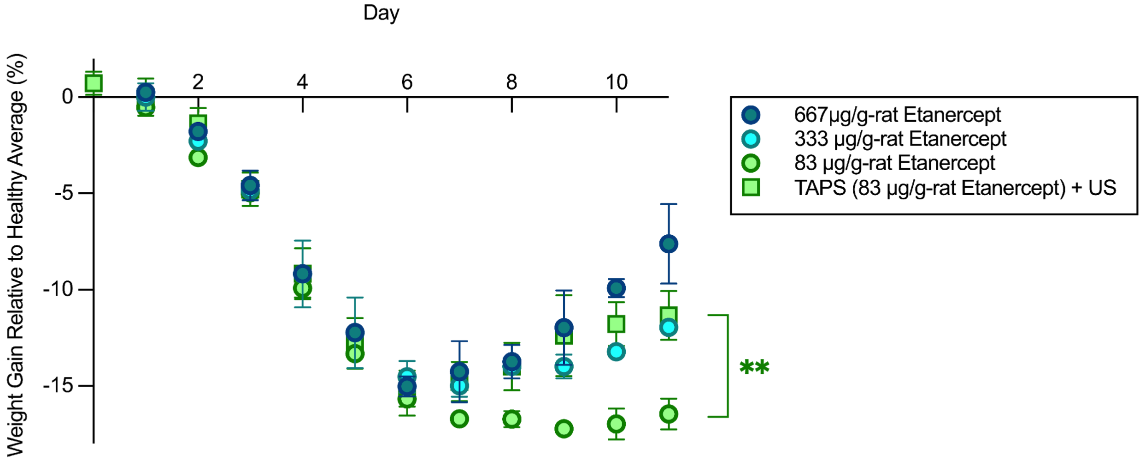

Supplementary Table 2 – Histopathology Assessment. Summary of histopathologist scores for the rat in vivo treatment of colitis with TAPS and Ultrasound (US) exposure

	**TAPS + US** (mean ± s.d., N = 6)	**TAPS** (mean ± s.d., N = 5)	**Control + US** (mean ± s.d., N = 5)	**Healthy + US** (mean ± s.d., N = 6)
**Inflammation severity** (none: 0, slight: 1, moderate: 2, and severe: 3)	0.8 ± 0.4	2.2 ± 0.8	2.4 ± 0.5	0.6 ± 0.7
**Polymorphonuclear Neutrophil (PMN) Infiltration / High Power Field (HPF)** (less than 5: 0, 5 to 20: 1, 21 to 60: 2, 61 to 100: 3, and more than 100: 4)	2.0 ± 0.6	3.2 ± 0.4	2.8 ± 0.8	1.7 ± 0.8
**Injury Depth** (none: 0, mucosa: 1, submucosa and mucosa: 2, and transmural: 3)	1.5 ± 0.5	2.2 ± 0.4	2.2 ± 0.4	0.6 ± 0.2
**Crypt Damage** (none: 0, basal 1/3: 1, basal 2/3: 2, only surface epithelium intact: 3, and total crypt lost: 4)	1.8 ± 1.1	3.0 ± 0.0	3.0 ± 1.0	0.4 ± 0.2

Supplementary Table 3 – List of histopathologist qualitative descriptions of rats in each experimental group

Group	Qualitative Observations
**TAPS + US**	Rat 1: Mild acute focal inflammation, goblet loss, atrophy, blunting<br>Rat 2: Moderate multifocal inflammation, acute, goblet loss mild, blunting and broadening<br>Rat 3: Moderate multifocal acute infiltration, goblet loss, atrophy, blunting<br>Rat 4: Moderate to severe acute multifocal inflammation, goblet loss, hyperplasia, blunting of villi<br>Rat 5: Very mild acute focal inflammation, minimal goblet loss, less neutrophil, mild broadening<br>Rat 6: Very mild acute inflammation, minimal goblet loss, almost normal structure
**TAPS**	Rat 1: Severe multifocal, acute inflammation, hyperplasia, goblet loss, blunting<br>Rat 2: Very severe acute multifocal inflammation, goblet and crypt loss, ulceration, blunting, necrosis<br>Rat 3: Severe multifocal acute inflammation, goblet loss, crypt loss, blunting, atrophied, ulceration<br>Rat 4: Severe acute multifocal inflammation., hyperplasia, Goblet cell loss, blunting of villi<br>Rat 5: Severe focal acute inflammation, goblet cell loss, crypt loss, blunting and broadening
**Control + US**	Rat 1: Very severe acute multifocal inflammation, abscess, goblet loss, blunting, atrophy<br>Rat 2: Acute multifocal inflammation, Goblet cell loss, hyperplasia, crypt loss, blunting, widening<br>Rat 3: Severe multifocal, inflammation, acute, severe goblet loss, ulceration, hyperplasia, blunting, crypt loss<br>Rat 4: Very severe multifocal inflammation, acute, Heavy goblet loss, marked hyperplasia, blunting, erosions<br>Rat 5: Very severe acute multifocal inflammation, abscesses, Goblet loss, blunting, broadening
**Healthy + US**	Rat 1: Moderate multifocal acute infiltration, goblet loss, atrophy, blunting<br>Rat 2: Very mild focal inflammation, minimal goblet loss, less neutrophil, mild broadening<br>Rat 3: Very mild acute multifocal inflammation, goblet loss, crypt loss mild, villi atrophied, mild blunting<br>Rat 4: Mild multifocal acute inflammation, neutrophils, hyperplasia, blunting, broadening<br>Rat 5: Mild multifocal acute inflammation, neutrophils, hyperplasia, blunting, broadening<br>Rat 6: Very mild acute inflammation, minimal goblet loss, almost normal structure
